# Can Sulfonylureas for Agricultural Use Cause Diabetes? A Report of Three Cases

**DOI:** 10.7759/cureus.35938

**Published:** 2023-03-09

**Authors:** Maria Kampouraki, Konstantina Mavridou, Maria Bakola, Konstantina Soultana Kitsou, Dimitris Karanasios

**Affiliations:** 1 Primary Care, Nea Madytos Health Center, Thessaloniki, GRC; 2 Department of Dermatology, University General Hospital of Ioannina, Ioannina, GRC; 3 Primary Care, Ioannina Health Center, Ioannina, GRC; 4 Department of Surgery, University General Hospital of Patras, Patras, GRC

**Keywords:** agronomists, herbicides, diabetes, agricultural use, sulfonylureas

## Abstract

Sulfonylureas (SUs) are commonly used as herbicides. Many farmers and other professionals use SUs for cereal, strawberry, and grape crops. This study examines the possible association between exposure to SUs herbicides and the risk of developing type 2 diabetes (T2D). The study presents three cases of unrelated agronomists who had used SUs for more than three decades and developed T2D. The objective was to investigate the association between occupational dermal and inhalation exposure to herbicides and T2D. Further studies with a larger sample size are needed to determine the association and to help develop prevention strategies.

## Introduction

Sulfonylureas (SUs) are a class of herbicides that inhibit the biosynthesis of branched-chain amino acids in plants by inhibiting the enzyme acetolactate synthase (ALS) [1,2]. These herbicides, such as tribenuron-methyl (TBM) and amidosulfuron, are widely used to grow cereals, strawberries, and grapes [3]. On the other hand, type 2 diabetes (T2D) is a complex and chronic disease with a strong genetic component, environmental factors, and lifestyle habits. Previous studies have indicated a possible association between exposure to herbicides, especially SUs, and the development of T2D. These studies have found that individuals exposed to SUs through their occupation or by living near areas where these herbicides are used are at higher risk of developing T2D. However, further research is needed to fully understand the mechanisms by which herbicides may contribute to the development of T2D and establish a definitive causal relationship [4-6]. We report three cases of agronomists who used SUs for up to three decades and developed T2D. These individuals used these herbicides regularly as part of their job duties.

## Case presentation

Three male agronomists, aged 48-55 years, presented to the primary care center with symptoms suggestive of T2D. Laboratory results for fasting blood glucose test [FBGT] (normal range 70-100 mg/dL), two-hour oral glucose tolerance test [OGTT (2h)] (normal <140 mg/dL), Hemoglobin A1c [HbA1c] (normal <6,5%), Lipid panel including total cholesterol [TC] (normal range 110-200 mg/dL), High-density lipoprotein cholesterol [HDL-C] (normal range 35-70 mg/dL), Low-density lipoprotein cholesterol [LDL-C] (normal <100 mg/dL) and triglyceride levels [TG] (normal range 40-175 mg/dL) personal and family history, and body mass index [BMI] (normal range 18.5 - 24.9 kg/m2) were collected for each subject and are shown in Table 1. All three individuals had used SUs in their work as agricultural scientists for up to three decades before T2D diagnosis.

**Table 1:** Summary of patients’ characteristics BMI: Body Mass Index; FBGT: Fasting Blood Glucose test; HbA1c: Hemoglobin A1c; OGTT (2h): 2-hour Oral Glucose Tolerance Test; TC: Total cholesterol; HDL-C: High-density lipoprotein cholesterol; LDL-C: Low-density lipoprotein cholesterol; TG: Triglyceride levels.

	Patient 1	Patient 2	Patient 3
Gender	male	male	male
Age	55	48	51
Personal/Family History	free	free	free
BMI kg/m2	24,3	23,6	22,8
Smoke/Alcohol Consumption	no	no	no
Exposure Duration (years)	30	20	27
FBGT mg/dL	130	155	215
HbA1c %	7	7,3	9
OGTT (2h) mg/dL	220	289	324
Lipid Panel (TC, HDL-C, LDL-C, TG) mg/dL	normal	normal	normal

Case 1

A 55-year-old male agronomist visited our family medicine center because of symptoms of fatigue and weight loss over the past six months. On examination and after taking a detailed history, he was found to be suffering from anorexia, especially with meat, and had lost 10 pounds during this period. He had been a farmer for 30 years and had used SUs to grow strawberries. Laboratory results showed the presence of diabetes mellitus. His HbA1c was 7.0%, FBGT was 130 mg/dL, and OGTT (2h) was 220 mg/dL. There was no history of diabetes mellitus in his family, and his BMI was 24.3 kg/m2. The other laboratory tests and imaging studies were normal.

Case 2

A 48-year-old agronomist visited our primary care center as part of his annual physical exam and had an FBGT of 155 mg/dL. Further laboratory testing revealed an HbA1c level of 7.3% and an OGTT (2h) of 289 mg/dL. He had been a farmer for 20 years and had used SUs for grain cultivation. There was no history of diabetes mellitus in his family, and his BMI was 23.6 kg/m2. The other laboratory tests and imaging studies were normal.

Case 3

The patient is a 51-year-old male agronomist who has been using SUs to grow grapes for 27 years. He presented with symptoms of excessive thirst and frequent urination, which were confirmed by laboratory tests to be due to diabetes mellitus (HbA1c: 9.0, FBGT: 215 mg/dL, OGTT 2h: 324 mg/dL). His family history is negative for diabetes, and his BMI is 22.8 kg/m2. Other tests were normal.

All patients were referred to a tertiary hospital and further evaluated by an endocrinologist. At the hospital, more specific laboratory and diagnostic imaging tests such as C-peptide, glutamic acid decarboxylase autoantibodies, and abdominal CT scans were performed, and the diagnosis of type 2 diabetes was confirmed. Patients were treated with oral antidiabetic drugs and are monitored by regular follow-ups to ensure the effectiveness of treatment.

## Discussion

Sulfonylurea compounds have a wide range of biological applications, including use as antidiabetics (e.g., glibenclamide), diuretics (e.g., torasemide), herbicides (e.g., chlorsulfuron), oncolytic (e.g., sulofenur), antimalarials, antifungals, and anticancer agents [7]. Agricultural workers may be exposed to various herbicides during activities such as transportation, mixing, application, or cleaning and maintenance of application equipment [8]. Due to the lack of information and adequate precautions, agricultural workers are at risk of a variety of chemical exposure-related manifestations, such as acute poisoning, blood, liver, and peripheral nervous system dysfunction, sleep apnea, Parkinson's disease, a higher incidence of rheumatoid arthritis, correlated infertility, and hypothyroidism [9]. T2D is a chronic disease characterized by chronic hyperglycemia resulting from inefficient use of insulin by the cells of the body. The number of people with diabetes worldwide has increased from 108 million in 1980 (4.7%) to 451 million in 2017 (8.5%). Moreover, the number of deaths due to diabetes has increased by 70% worldwide between 2000 and 2019 [10]. It is well known that a healthy diet low in saturated fat, sweet products, and high-fiber foods, regular physical activity, maintaining an average weight, and abstaining from smoking are well-known strategies to prevent or delay the onset of T2D. However, recent research suggests that environmental exposures such as herbicides may also play a role in the development of T2D. Studies have found an association between exposure to certain herbicides and an increased risk of T2D, suggesting that exposure to these chemicals may contribute to the development of this disease [8,10,11]. Previous publications have suggested that there may be a possible association between exposure to pesticides and the manifestation of diabetes. These studies have suggested several potential mechanisms through which pesticides may contribute to the development of diabetes, including insulin resistance, abnormal glucose regulation, oxidative stress, and the development of metabolic syndrome [4-6,8]. Pesticides can affect biological functions through several mechanisms, including epigenetic modulation that alters DNA methylation, histone modification, and microRNA expression. The effects of BPA, for example, can be long-lasting and are often mediated by epigenetic mechanisms such as DNA methylation. Epigenetic changes do not alter the DNA sequence but can lead to long-term changes in gene expression and cellular function. Although the study of epigenetics and its relationship to metabolic diseases is still a relatively new field, it is gaining attention and growing rapidly. Factors such as environment, lifestyle, diet, or gut microbiota can influence epigenetic programming during gamete formation, fetal and early postnatal development, and throughout life [11]. In this study, we present three cases of agricultural workers who were exposed to SUs for at least 20 years and developed T2D, although they were healthy, did not smoke, and had normal BMI. Our results suggest that chronic occupational exposure to herbicides containing SUs compounds, in combination with inadequate protective measures, may act as metabolic disruptors and contribute to the development of T2D. Our article has several limitations. First, the study included only three male patients who were not selected from a representative population sample. Therefore, the small sample size limits our ability to generalize and establish valid statistical relationships. Second, we did not perform toxicological testing. However, we were able to refer to a published Centers for Disease Control and Prevention (CDC) document that states that the human health effects of low-level environmental exposure to sulfonylurea herbicides remain unknown. While studies in mammals indicate that these herbicides are relatively nontoxic, high doses can cause nonspecific effects such as weight loss and anemia. Overall, the effects of biologically monitored levels of sulfonylurea herbicides on human health are not fully known [12]. In our article on three cases, we found no evidence of chronic toxicity. It is important to note that these three cases do not prove a causal relationship between SUs' herbicide exposure and T2D. However, the fact that all three individuals were exposed to SUs herbicides over a prolonged period and developed T2D despite being otherwise healthy is intriguing and warrants further investigation. Further studies with larger samples are needed to better understand the relationship between SUs herbicides and diabetes. In the meantime, appropriate protective equipment, safety precautions, and handling procedures should be used when working with these substances to minimize the risk of exposure.

## Conclusions

Our case reports suggest a possible association between long-term exposure to SUs and the development of T2D. The cases presented in this study were agronomists who had been exposed to SUs compounds for at least 20 years through their occupation. The possible mechanism of action could be the ingestion of SUs compounds through the skin or through inhalation, which affects the cells of the pancreas and disrupts proper insulin production, eventually leading to T2D. It is important to note that these cases are not definitive proof of a causal relationship between exposure to SUs and T2D, but they suggest a possible correlation that warrants further investigation. In addition, these cases highlight the need for further research on the long-term health effects of exposure to these herbicides and the need to be cautious in their use. It is also important to note that farmers, farm workers, and others who regularly use these herbicides should take appropriate precautions to minimize their exposure and watch for signs of T2D or other health problems.
